# EQ-5D-Y-3L population norms for children in Mainland China derived from a national survey 2023–2024

**DOI:** 10.1186/s12955-025-02470-z

**Published:** 2025-12-29

**Authors:** Wenwen Chen, Yibo Wu, Xiaodan Zhang, Jiale Qi, Jingbo He, Xiaolin Xu, Yanping He, Hongyan Yang, Chaojie Liu, Qiang Yao

**Affiliations:** 1 Centre for Social Security Studies, Wuhan University, Wuhan, Hubei China; 2 School of Political Science and Public Administration, Wuhan University, Wuhan, Hubei China; 3 Department of Nursing, the Fourth Affiliated Hospital of School of Medicine and International School of Medicine, International Institutes of Medicine, Zhejiang University, Yiwu, 322000 China; 4 College of Media and International Culture, Zhejiang University, Hangzhou, China; 5 School of Public Health, Shanxi Medical University, Taiyuan, Shanxi 030001 China; 6 National Center for Children’s Health, Beijing Children’s Hospital, Capital Medical University, Beijing, China; 7 School of Psychology and Public Health, La Trobe University, Melbourne, VIC Australia

**Keywords:** EQ-5D-Y-3L, Population norms, China, HRQoL

## Abstract

**Background:**

The EQ-5D-Y-3L and value sets have been developed in mainland China. However, a nationally representative EQ-5D-Y-3L population norm for children has not yet been established. This study aims to develop EQ-5D-Y-3L norms for children in mainland China based on a national survey.

**Methods:**

The data for this study were derived from the Medication Literacy Investigation of Chinese Children (MLICC). The survey spanned 18 geographically and socioeconomically diverse provinces/regions in mainland China, employing a multi-stage quota sampling strategy stratified by gender and age. Child participants (8–18 years) completed the survey through an online platform, while guardians provided household information. The survey was supervised by trained investigators through one-on-one, face-to-face interviews, yielding a final sample of 5,191 participants for analysis. The utility index (UI) was reported for the entire sample (weighted using 2020 census data) and by the characteristics of the study participants.

**Results:**

The weighted mean UI score was 0.951 (SD = 0.099). The most frequently reported problem was feeling worried, sad or unhappy (WSU) (24.59%), followed by having pain or discomfort (PD) (17.87%), doing usual activities (UA) (7.73%), and mobility (MO) (4.76%), with looking after myself (LAM) being the least reported problem (4.23%). Respondents who were male, younger, and those with better behaviours, no chronic conditions, better family functioning, and residence in a relatively economically developed region had higher UI scores (*p* < 0.001).

**Conclusions:**

This study provides national EQ-5D-Y-3L population norms for children in mainland China. The study found that children in mainland China have relatively higher UI scores compared with those in other countries. A wide range of socioeconomic and behavioral factors are associated with the UI scores.

**Trial registration:**

Clinical trial number was not applicable.

**Supplementary Information:**

The online version contains supplementary material available at 10.1186/s12955-025-02470-z.

## Background

Health-related quality of life (HRQoL) studies on children have increased over the past two decades [1]. HRQoL measures individuals’ perceived well-being across physical, mental, and social health domains [2]. These measures are valuable for assessing population health status and evaluating the cost-effectiveness of healthcare interventions [3]. However, most HRQoL instruments have been developed for adult populations, which are not always applicable to child populations due to differences in life stage and health and wellbeing needs [4–6]. Consequently, research on children’s HRQoL warrants special attention [7] to capture broader outcomes and provide a comprehensive understanding of their health [8].

A recent review identified 15 generic HRQoL instruments for children [9]. Among these, the EQ-5D-Y-3 L has been widely employed in individuals aged 8–15 years [10]. The child-friendly EQ-5D version, known as EQ-5D-Y-3 L (formerly EQ-5D-Y), was introduced by the EuroQol Group in 2009 as a more comprehensible instrument suitable for children and adolescents. The instrument has undergone rigorous psychometric evaluation, demonstrating strong validity, reliability, and responsiveness in diverse child populations with differing health conditions [11].

The EQ-5D-Y-3 L encompasses five dimensions, each rated on three levels [10]. A utility index (UI) score is derived from a combination of these five dimensions using population preference value sets. Population norms are essential for interpreting UI results [12, 13]. As a benchmarking tool, population norms facilitate HRQoL comparisons and support health economic studies, pharmacoeconomic evaluations, and public health decision-making processes. Several countries have developed EQ-5D-Y-3 L norms, such as Japan [14], Peru [15], Chile [16], Indonesia [17] and Australia [18], which play a pivotal role in identifying health disparities, monitoring progress toward health goals, and informing public health policies.

The EQ-5D-Y-3 L and its value sets have been developed in mainland China [19], and have been utilized to measure HRQoL in children with hematological malignancies [20]. However, a nationally representative EQ-5D-Y-3 L population norm for children has not yet been established. This absence of reference values limits the interpretation of EQ-5D-Y-3 L results, impedes the calculation of quality-adjusted life years (QALYs) for children, and restricts the broader application of EQ-5D-Y-3 L in pediatric studies in China.

This study aims to develop EQ-5D-Y-3L norms for children in mainland China based on a national survey. This effort will address current gaps in China’s EQ-5D methodological framework and play a critical role in promoting and standardizing the use of EQ-5D-Y-3L in health surveys, policy evaluations, economic assessments, and clinical trial research involving children.

## Data and methods

### Study design and data collection

The data for this study were derived from the Medication Literacy Investigation of Chinese Children (MLICC), a cross-sectional survey conducted from October 2023 to May 2024. The MLICC was initiated, designed and implemented by Beijing Children’s Hospital, Capital Medical University, spanning 18 geographically and socioeconomically diverse provinces/regions in mainland China. It collected information on respondents’ physical function, behaviours, family circumstances, and health, including EQ-5D-Y-3L data.

MLICC employed a multi-stage quota sampling strategy. In the first stage, 18 provinces/regions were selected from China’s eastern, central, and western economic zones to ensure representativeness. Subsequently, within each province/region, the capital city and 2–6 prefecture-level cities were chosen using random numbers. A quota was allocated to each city based on the 2020 China Population Census, maintaining a 1:1 gender ratio and an age distribution consistent with the census data. The total target sample size was 5,000 participants.

Study participants were recruited using multiple strategies, such as posters, social media notices, and site visits, across various settings including local schools, residential communities, and hospitals. Trained investigators approached parents or guardians at the recruitment sites to obtain consent for the child’s participation. Once the parents or guardians agreed, the investigators confirmed the child’s willingness to participate and then obtained written informed consent from the parents or guardians for both the child and themselves.

The survey was conducted through face-to-face interviews at locations (home, school, hospital, or community) convenient for the participants. The investigators, who were health workers from children’s hospitals (ranging from one to six per province/region), were required to demonstrate strong communication and teamwork skills and had to complete and pass a standardized training session. The investigators were trained to adhere to a strict protocol. They explained the purpose of the survey and how to complete it, provided clarification regarding the meaning of questions if requested, but did not influence respondents’ answers. All data were entered into the online platform Wenjuanxing (https://www.wjx.cn/). Child respondents were asked to complete the questionnaire independently at first. They completed questions relating to their own experiences, such as physical and mental functioning, on their own. However, they were likely to seek advice from their parents/guardians when answering questions related to income and other household matters.

The inclusion criteria for participation in this study were as follows: (1) aged between 8 and 18 years; (2) both the children and their guardians voluntarily agreed to participate, with guardians providing informed consent; (3) the children were Chinese citizens; (4) the children lived permanently in local households, spending no more than one month away annually; (5) the children could complete the questionnaire independently or with the assistance of a guardian.

A total of 5,668 potential child participants were approached, and 103 declined the invitation, resulting in 5,565 returned questionnaires. Field audits and logical checks excluded 173 responses due to ineligible age and 201 due to logical inconsistencies. This resulted in a final sample of 5,191 valid responses for this study.

### Measurements

The MLICC survey collected comprehensive data on participants’ demographic characteristics, family status, psychological development, health and health behaviours (including EQ-5D-Y-3L), living environment, and attitudes towards prominent social issues. The EQ-5D-Y-3L was administered in the middle section of the survey.

#### Outcome indicators

Two types of indicators were used: the percentage of reported problems in each dimension and the UI scores.

The EQ-5D-Y-3 L assessed problems in mobility (MO), looking after myself (LAM), doing usual activities (UA), having pain or discomfort (PD), and feeling worried, sad or unhappy (WSU) using a three-point Likert scale, ranging from 1 (“no problem”) to 3 (“a lot of problems”) [21]. The percentage of respondents reporting problems (scoring 2 or 3) in each dimension was calculated.

UI scores were derived for each respondent using the Chinese children value sets developed by Yang et al. [19]. The scores were calculated using the formula below, ranging from − 0.089 (worst) to 0.977 (best):

Utility = 1-0.050*MO2-0.183*MO3-0.011*LAM2-0.127*LAM3-0.046*UA2-0.170*UA3-0.078*PD2-0.267*PD3-0.061*WSU2-0.172*WSU3-0.012-0.158*A3.

MO2/LAM2/UA2/PD2/WSU2: A score of 2 in this dimension, that is, “some problems”.

MO3/LAM3/UA3/PD3/WSU3: A score of 3 in this dimension, that is, “a lot of problems”.

#### Sociodemographic characteristics

HRQoL has been shown to vary based on demographic characteristics [22, 23], health risk behaviours [24, 25], health status [26, 27], socioeconomic status [28, 29], and family status [30, 31]. This study reports EQ-5D-Y-3 L results categorized by these characteristics:

- **Demographic characteristics**: Gender (male or female) and age (8–11, 12–15, 16–18 years).
- **Behaviours**: Smoking and drinking in the past 12 months (yes or no), daily sleep time (< 6, 6–7, > 7 h), and sleep quality (very poor, poor, good, very good) measured using one item chosen from the Sleep Quality Scale (B-PSQI) [32, 33].
- **Health status**: Chronic conditions (yes or no) measured by a checklist and BMI (moderate to severe weight loss, mild weight loss, normal, overweight and obesity) [34, 35].
- **Socioeconomic status**: Residence (urban or rural) and economic zone (eastern developed, central developing, western underdeveloped) [36].
- **Family status**: Living arrangements (living with both parents, not living with parents, living with one parent), average family contact per day (0–7, 8–15, 16–24 h), presence of siblings (yes or no), and family functioning defined as “the extent to which family members are able to resolve problems and communicate effectively and ensure proper role allocation, emotional response and involvement within the family” [37] (poor [< 25th percentile], moderate [25th-75th percentile], excellent [> 75th percentile] based on the current study sample) measured by the Family Health Scale (FHS-SF) [38].

### Statistical analysis

Frequency distributions and means (with standard deviations) were used to describe nominal/ordinal and continuous variables, respectively. Since UI scores were not normally distributed, their medians (50th percentile) and interquartile ranges (25th and 75th percentiles) are also reported. Population scores were presented by age, gender, and residence, with weights calculated based on the seventh Chinese Population Census (see Appendix Table 1).

**Table 1:** Sociodemographic characteristics of respondents

Variable	*N*	%	*N* (weighted)	% (weighted)
**Demographic characteristics**				
Gender				
Male	2364	45.54	2770	53.37
Female	2827	54.46	2421	46.63
Age (years)				
8–11	1104	21.27	1945	37.47
12–15	2077	40.01	1788	34.45
16–18	2010	38.72	1458	28.08
**Behaviour**				
Smoking				
No	4920	94.78	4927	94.92
Yes	271	5.22	264	5.08
Drinking				
No	4111	79.19	4285	82.54
Yes	1080	20.81	906	17.46
Sleep time (hours)				
< 6	396	7.63	334	6.44
6–7	1379	26.57	1143	22.01
> 7	3416	65.81	3714	71.55
Sleep quality				
Very poor	161	3.1	155	2.99
Poor	790	15.22	673	12.97
Good	2565	49.41	2475	47.68
Very good	1675	32.27	1887	36.36
**Health**				
Chronic conditions				
No	4695	90.45	4668	89.93
Yes	496	9.55	523	10.07
BMI				
Moderate to severe weight loss	247	4.76	244	4.71
Mild weight loss	213	4.10	215	4.14
Normal	2635	50.76	2543	48.99
Overweight	536	10.33	565	10.89
Obesity	1560	30.05	1623	31.26
**Family status**				
Living arrangement				
Living with both parents	3824	73.67	3890	74.94
Not living with parents	615	11.85	586	11.28
Living with one parent	752	14.49	715	13.77
Average family contact per day (hours)				
0–7	865	16.66	793	15.27
8–15	2766	53.28	2756	53.09
16–24	1560	30.05	1642	31.64
Having siblings				
No	1593	30.69	1529	29.46
Yes	3598	69.31	3662	70.54
Family functioning				
Poor	1091	21.02	1118	21.54
Moderate	2220	42.77	2221	42.79
Excellent	1880	36.22	1851	35.66
**Socioeconomic status**				
Residence				
Urban	3490	67.23	2951	56.84
Rural	1701	32.77	2240	43.16
Region				
Eastern developed	1753	33.77	1742	33.56
Central developing	2281	43.94	2377	45.80
Western underdeveloped	1157	22.29	1071	20.64
**Overall**	5191		5191	

Chi-square tests were used to compare the percentages of respondents reporting problems across different characteristics. Logistic regression models identified sociodemographic factors associated with reporting problems, presenting adjusted odds ratios (AORs) and 95% confidence intervals (CIs).

Differences in UI scores among respondents were assessed using ANOVA. Tobit regression models identified predictors of UI scores, presenting standardized regression coefficients with 95% CIs calculated using robust standard errors. The Tobit regression model is commonly used for truncated outcome variables in econometric researches [39]. Because our outcome metric (EQ-5D UI score) is strictly bilaterally truncated, the Tobit model is well suited [40].

A p-value less than 0.05 was considered statistically significant. All analyses were conducted using STATA version 16.0 (SE) for Windows.

### Ethical statement

The MLICC study received ethical approval from the Ethics Committee of Beijing Children’s Hospital, Capital Medical University ([2023]-E-015-R). The survey adhered to the ethical principles for biomedical research involving humans, as outlined in the World Medical Association’s Declaration of Helsinki and the International Ethical Guidelines for Human Biomedical Research.

## Results

### Characteristics of respondents

Compared to the 2020 census data, our sample was biased toward urban female residents, while rural males were underrepresented. More than half of the respondents were female (54.46%), and the largest age group was those aged 12 to 15 years (40.01%). Most respondents did not smoke (94.78%) or drink alcohol (79.19%) in the past year and reported no chronic conditions (90.45%). Nearly two-thirds slept more than seven hours per night (65.81%), and most rated their sleep quality as good (49.41%) and very good (32.27%). Approximately half of respondents (50.76%) had a normal BMI. Most respondents lived with both parents (73.67%), had siblings (69.31%), resided in urban areas (67.23%), and spent an average of 8 to 15 h with their family daily (53.28%). Two-fifths reported moderate family functioning (42.77%). The central zone accounted for the largest proportion of respondents (43.94%) (Table 1).

### Percentage of reported health problems

Overall, the weighted proportion of respondents who reported no problems in all dimensions (status 11111) was 65.38%, while approximately one-third (34.62%) reported having problems (Table 2). The most frequently reported problem was feeling worried, sad or unhappy (WSU) (24.59%), followed by having pain or discomfort (PD) (17.87%), doing usual activities (UA) (7.73%), and mobility (MO) (4.76%), with looking after myself (LAM) being the least reported problem (4.23%).

**Table 2:** Percentage of respondents reporting health problems and EQ-5D-Y-3L utility index (weighted)

Variable	Mobility	Looking after myself	Doing usual activities	Having pain or discomfort	Feeling worried, sad or unhappy	Any problem	EQ-5D-Y-3L Utility Index (Weighted)			
	*N* = 247	*N* = 220	*N* = 401	*N* = 928	*N* = 1276	*N* = 1797	Mean	SD	Median	IQR
**Gender**	*p = 0.553*	*p = 0.059*	***p*** ** < 0.001**	***p*** ** < 0.001**	*p = 0.918*	***p*** ** < 0.001**	***p*** ** < 0.001**			
Male	6.03%	4.93%	8.09%	16.43%	21.48%	32.22%	0.953	0.102	1.000	0.073
Female	3.30%	3.44%	7.33%	19.52%	28.14%	37.36%	0.949	0.096	1.000	0.073
**Age (years)**	***p*** ** < 0.001**	***p*** ** < 0.001**	***p*** ** < 0.001**	***p*** ** < 0.001**	***p*** ** < 0.001**	***p*** ** < 0.001**	***p*** ** < 0.001**			
8–11	4.90%	6.41%	7.06%	13.18%	17.33%	28.19%	0.959	0.099	1.000	0.058
12–15	4.45%	3.22%	7.93%	19.53%	26.49%	36.43%	0.948	0.101	1.000	0.073
16–18	4.95%	2.57%	8.40%	22.10%	31.94%	40.95%	0.943	0.096	1.000	0.090
**Smoking**	***p*** ** < 0.001**	***p*** ** < 0.001**	***p*** ** < 0.001**	***p*** ** < 0.001**	***p*** ** < 0.001**	***p*** ** < 0.001**	***p*** ** < 0.001**			
No	4.44%	4.13%	7.47%	16.96%	23.57%	33.57%	0.954	0.093	1.000	0.073
Yes	10.84%	6.21%	12.66%	34.82%	43.58%	54.22%	0.894	0.166	0.927	0.151
**Drinking**	***p*** ** < 0.001**	***p*** ** = 0.035**	*p = 0.040*	*p* = 0.883	***p*** ** < 0.001**	***p*** ** < 0.001**	***p*** ** < 0.001**			
No	4.55%	4.33%	7.27%	15.03%	20.32%	30.42%	0.958	0.094	1.000	0.073
Yes	5.74%	3.76%	9.92%	31.31%	44.75%	54.46%	0.918	0.115	0.927	0.151
**Sleep time (hours)**	***p*** ** < 0.001**	***p*** ** < 0.001**	***p*** ** < 0.001**	***p*** ** < 0.001**	***p*** ** < 0.001**	***p*** ** < 0.001**	***p*** ** < 0.001**			
< 6	10.08%	4.52%	12.69%	30.51%	39.92%	51.02%	0.911	0.132	0.942	0.151
6–7	4.58%	2.76%	7.13%	23.04%	31.22%	41.14%	0.945	0.088	1.000	0.090
> 7	4.34%	4.66%	7.47%	15.14%	21.16%	31.13%	0.956	0.098	1.000	0.073
**Sleep quality**	***p*** ** < 0.001**	*p* = 0.003	***p*** ** < 0.001**	***p*** ** < 0.001**	***p*** ** < 0.001**	***p*** ** < 0.001**	***p*** ** < 0.001**			
Very poor	12.65%	7.83%	18.96%	37.32%	43.64%	54.77%	0.871	0.192	0.927	0.184
Poor	7.67%	4.36%	12.28%	35.27%	52.69%	62.65%	0.908	0.107	0.927	0.151
Good	4.23%	3.97%	7.65%	18.04%	25.01%	35.40%	0.952	0.094	1.000	0.073
Very good	3.77%	4.24%	5.30%	9.84%	12.43%	21.93%	0.971	0.081	1.000	0.000
**Chronic conditions**	***p*** ** < 0.001**	***p*** ** < 0.001**	***p*** ** < 0.001**	***p*** ** < 0.001**	***p*** ** < 0.001**	***p*** ** < 0.001**	***p*** ** < 0.001**			
No	3.87%	3.82%	6.82%	15.12%	21.08%	30.78%	0.959	0.085	1.000	0.073
Yes	12.72%	7.95%	15.90%	42.41%	55.89%	68.83%	0.874	0.161	0.927	0.151
**BMI**	***p*** ** < 0.001**	***p*** ** < 0.001**	***p*** ** < 0.001**	***p*** ** < 0.001**	***p*** ** < 0.001**	***p*** ** < 0.001**	***p*** ** < 0.001**			
Moderate to severe weight loss	7.26%	3.86%	5.48%	22.63%	25.73%	36.39%	0.944	0.095	1.000	0.090
Mild weight loss	5.59%	4.68%	7.69%	18.20%	26.52%	37.77%	0.944	0.109	1.000	0.073
Normal	4.90%	4.03%	6.83%	17.55%	24.69%	34.06%	0.950	0.106	1.000	0.073
Overweight	5.86%	4.31%	8.08%	17.60%	22.30%	31.04%	0.953	0.094	1.000	0.073
Obesity	3.68%	4.53%	9.37%	17.71%	24.78%	36.05%	0.953	0.088	1.000	0.073
**Living arrangement**	***p*** ** < 0.001**	*p = 0.844*	***p*** ** < 0.001**	***p*** ** < 0.001**	***p*** ** < 0.001**	***p*** ** < 0.001**	***p*** ** < 0.001**			
Living with both parents	4.30%	3.79%	7.06%	16.11%	22.20%	31.60%	0.957	0.092	1.000	0.073
Not living with parents	6.67%	6.10%	10.65%	23.93%	32.60%	46.26%	0.932	0.107	1.000	0.090
Living with one parent	5.69%	5.12%	9.04%	22.51%	31.00%	41.48%	0.935	0.123	1.000	0.090
**Average family contact per day(hours)**	***p*** ** < 0.001**	*p = 0.259*	***p*** ** < 0.001**	***p*** ** < 0.001**	***p*** ** < 0.001**	***p*** ** < 0.001**	***p*** ** < 0.001**			
0–7	5.67%	4.17%	8.91%	22.98%	28.54%	39.87%	0.941	0.103	1.000	0.090
8–15	4.16%	3.66%	7.91%	17.83%	25.56%	35.01%	0.952	0.092	1.000	0.073
16–24	5.34%	5.23%	6.87%	15.48%	21.04%	31.42%	0.953	0.107	1.000	0.073
**Having siblings**	*p = 0.057*	***p*** ** < 0.001**	***p*** ** < 0.001**	*p* = 0.205	***p*** ** < 0.001**	***p*** ** < 0.001**	***p*** ** < 0.001**			
No	4.46%	4.90%	6.08%	14.90%	20.96%	30.38%	0.958	0.093	1.000	0.073
Yes	4.89%	3.95%	8.42%	19.11%	26.10%	36.38%	0.948	0.101	1.000	0.073
**Family functioning**	*p = 0.096*	***p*** ** < 0.001**	***p*** ** < 0.001**	***p*** ** < 0.001**	***p*** ** < 0.001**	***p*** ** < 0.001**	***p*** ** < 0.001**			
Poor	7.56%	5.73%	11.75%	25.19%	34.69%	45.20%	0.927	0.120	1.000	0.119
Moderate	5.09%	4.88%	8.60%	19.64%	25.67%	36.76%	0.946	0.109	1.000	0.073
Excellent	2.67%	2.56%	4.27%	11.32%	17.18%	25.65%	0.971	0.060	1.000	0.023
**Residence**	*p = 0.070*	***p*** ** < 0.001**	***p*** ** < 0.001**	***p*** ** < 0.001**	***p*** ** < 0.001**	***p*** ** < 0.001**	***p*** ** < 0.001**			
Urban	3.12%	3.45%	6.54%	16.75%	23.64%	33.09%	0.957	0.088	1.000	0.073
Rural	6.93%	5.27%	9.31%	19.35%	25.84%	36.63%	0.943	0.111	1.000	0.073
**Region**	***p*** ** < 0.001**	***p*** ** < 0.001**	*p* = 000	***p*** ** < 0.001**	***p*** ** < 0.001**	***p*** ** < 0.001**	***p*** ** < 0.001**			
Eastern developed	4.22%	3.57%	5.72%	14.56%	18.80%	27.94%	0.963	0.078	1.000	0.069
Central developing	5.22%	4.44%	7.40%	18.46%	25.29%	34.84%	0.946	0.113	1.000	0.073
Western underdeveloped	4.62%	4.85%	11.74%	21.95%	32.43%	44.96%	0.941	0.094	1.000	0.090
**Overall**	4.76%	4.23%	7.73%	17.87%	24.59%	34.62%	0.951	0.099	1.000	0.073

The multivariable logistic regression models indicated that the odds of reporting problems varied based on respondents’ sociodemographic characteristics (Table 3). Specifically, children with chronic conditions had higher odds of reporting problems in all five dimensions (*p < 0.05*). Female gender was associated with lower odds of reporting problems in MO (*p < 0.001*), but higher odds of reporting problems in PD (*p < 0.05*) and WSU (*p < 0.001*). The odds of reporting LAM problems decreased with age (compared to those aged 8–11 years): 12–15 years (*p < 0.001*) and 16–18 years (*p < 0.001*). However, respondents aged 12–15 years had higher odds of reporting problems in PD (*p < 0.05*) and WSU (*p < 0.01*) than their younger counterparts.

**Table 3:** Factors associated with the percentage of health problems reported by respondents - results of logistic regression models (weighted)

	Mobility		Looking after myself		Doing usual activities		Having pain or discomfort		Feeling worried, sad or unhappy	
**Variable**	AOR	95%CI	AOR	95%CI	AOR	95%CI	AOR	95%CI	AOR	95%CI
**Gender**										
Ref: Male										
Female	0.530^***^	(0.372,0.755)	0.717	(0.485,1.062)	0.869	(0.664,1.138)	1.223^*^	(1.016,1.474)	1.433^***^	(1.210,1.696)
**Age (years)**										
Ref: 8–11										
12–15	0.917	(0.587,1.434)	0.454^***^	(0.292,0.707)	1.055	(0.743,1.496)	1.338^*^	(1.024,1.749)	1.407^**^	(1.113,1.779)
16–18	1.001	(0.596,1.680)	0.349^***^	(0.204,0.595)	1.001	(0.676,1.482)	1.121	(0.845,1.489)	1.261	(0.980,1.623)
**Smoking**										
Ref: No										
Yes	2.090^*^	(1.101,3.968)	1.516	(0.691,3.324)	1.096	(0.642,1.870)	1.171	(0.817,1.678)	0.881	(0.624,1.244)
**Drinking**										
Ref: No										
Yes	0.595^*^	(0.372,0.952)	0.812	(0.470,1.403)	0.930	(0.675,1.281)	1.651^***^	(1.333,2.045)	2.152^***^	(1.763,2.627)
**Sleep time(hours)**										
Ref: <6										
6–7	0.534^*^	(0.301,0.950)	0.778	(0.371,1.632)	0.727	(0.479,1.102)	0.878	(0.657,1.174)	0.867	(0.643,1.168)
> 7	0.485^*^	(0.268,0.879)	0.986	(0.502,1.934)	0.891	(0.603,1.318)	0.733^*^	(0.549,0.979)	0.821	(0.608,1.108)
**Sleep quality**										
Ref: Very poor										
poor	0.830	(0.428,1.611)	0.686	(0.300,1.570)	0.589	(0.344,1.009)	0.906	(0.550,1.493)	1.394	(0.868,2.238)
good	0.564	(0.291,1.095)	0.616	(0.285,1.329)	0.440^***^	(0.268,0.724)	0.519^**^	(0.316,0.852)	0.568^**^	(0.356,0.906)
Very good	0.500^*^	(0.251,0.998)	0.555	(0.251,1.229)	0.322^***^	(0.185,0.560)	0.312^***^	(0.185,0.526)	0.299^***^	(0.184,0.487)
**Chronic conditions**										
Ref: No										
Yes	3.056^***^	(1.892,4.939)	1.787^*^	(1.041,3.066)	2.036^***^	(1.371.3.023)	3.271^***^	(2.499,4.281)	3.775^***^	(2.867,4.971)
**BMI**										
Ref: Normal										
Moderate to severe weight loss	1.170	(0.595,2.300)	1.223	(0.582,2.570)	1.121	(0.647,1.944)	1.137	(0.752,1.717)	1.179	(0.809,1.718)
Mild weight loss	1.580	(0.773,3.228)	0.802	(0.296,2.173)	0.825	(0.462,1.472)	1.626^*^	(1.011,2.613)	1.265	(0.816,1.960)
Overweight	1.183	(0.649,2.157)	1.041	(0.558,1.940)	1.343	(0.821,2.198)	1.162	(0.834,1.619)	1.048	(0.798,1.377)
Obesity	0.744	(0.497,1.114)	1.075	(0.724,1.594)	1.422^*^	(1.071,1.890)	1.160	(0.943,1.428)	1.168	(0.965,1.413)
**Living arrangement**										
Ref: Living with both parents										
Not living with parents	1.063	(0.640,1.763)	1.534	(0.833,2.827)	1.138	(0.776,1.670)	1.095	(0.800,1.498)	1.133	(0.854,1.505)
Living with one parent	0.971	(0.639,1.476)	1.299	(0.786,2.147)	1.044	(0.721.1.513)	1.099	(0.859,1.405)	1.141	(0.906,1.437)
**Average family contact per day(hours)**										
Ref: 0–7										
8–15	0.965	(0.600,1.553)	0.844	(0.499,1.425)	1.064	(0.767,1.475)	0.955	(0.740,1.231)	1.205	(0.968.1.501)
16–24	1.384	(0.827,2.316)	1.279	(0.726,2.255)	0.954	(0.659,1.380)	0.913	(0.685,1.217)	1.059	(0.827,1.357)
**Having siblings**										
Ref: No										
Yes	0.999	(0.701,1.423)	0.692^*^	(0.479,1.000)	1.288	(0.958,1.731)	1.307^**^	(1.068,1.598)	1.223^*^	(1.013,1.477)
**Family functioning**										
Ref: Poor										
Moderate	0.801	(0.543,1.181)	0.957	(0.618,1.481)	0.827	(0.616,1.111)	0.950	(0.758,1.191)	0.835	(0.680,1.026)
Excellent	0.491^**^	(0.297,0.811)	0.521^*^	(0.300,0.903)	0.453^***^	(0.306,0.671)	0.600^***^	(0.469,0.767)	0.616^***^	(0.493,0.770)
**Residence**										
Ref: Urban										
Rural	0.480^***^	(0.342,0.674)	0.774	(0.521,1.150)	0.895	(0.681,1.178)	1.034	(0.854,1.253)	1.139	(0.954,1.360)
**Region**										
Ref: Eastern developed										
Central developing	0.903	(0.586,1.393)	1.001	(0.644,1.558)	1.101	(0.791,1.532)	1.120	(0.910,1.377)	1.308^**^	(1.084,1.576)
Western underdeveloped	0.744	(0.446,1.242)	1.320	(0.745,2.339)	1.593^*^	(1.074,2.364)	1.062	(0.811.1.390)	1.354^**^	(1.077,1.702)

*: P-value < 0.05, **: P-value < 0.01, ***: P-value < 0.001, AOR: adjusted odds ratio, CI: 95% confidence interval

Smoking was associated with higher odds of reporting problems in MO (*p < 0.05*). Alcohol consumption was linked to lower odds of reporting problems in MO (*p < 0.05*), but higher odds of reporting problems in PD and WSU (*p < 0.001*).

Longer sleep duration (compared to < 6 h) was associated with lower odds of reporting problems in MO (AOR = 0.534 for 6–7 h, *p* < 0.05; AOR = 0.485 for > 7 h, *p* < 0.05) and PD (AOR = 0.733 for > 7 h, *p* < 0.05). Higher sleep quality (compared to very poor or poor) was linked to lower odds of reporting problems in MO (AOR = 0.500 for very good, *p* < 0.05), UA (AOR = 0.440 for good, *p* < 0.001; AOR = 0.322 for very good, *p* < 0.001), PD (AOR = 0.519 for good, *p* < 0.01; AOR = 0.312 for very good, *p* < 0.001), and WSU (AOR = 0.568 for good, *p* < 0.01; AOR = 0.299 for very good, *p* < 0.001).

Obesity was associated with higher odds of reporting problems in UA (AOR = 1.422, *p* < 0.05) while mild weight loss was associated with higher odds of reporting problems in PD (AOR = 1.626, *p* < 0.05) compared to those with a normal BMI. Having siblings was linked to lower odds of reporting problems in LAM (AOR = 0.692, *p* < 0.05) but higher odds of reporting problems in PD (AOR = 1.307, *p* < 0.01) and WSU (AOR = 1.223, *p* < 0.05).

Excellent family functioning (compared to poor) was associated with lower odds of reporting problems in MO (AOR = 0.491, *p* < 0.01), LAM (AOR = 0.521, *p* < 0.05), UA (AOR = 0.453, *p* < 0.001), PD (AOR = 0.600, *p* < 0.001), and WSU (AOR = 0.616, *p* < 0.001). Rural residency was associated with lower odds of reporting problems in MO (AOR = 0.480, *p* < 0.001).

Respondents living in the underdeveloped western zone had higher odds of reporting problems in UA (AOR = 1.593, *p* < 0.05) and WSU (AOR = 1.354, *p* < 0.01), while those living in the central developing zone had higher odds of reporting problems in WSU (AOR = 1.308, *p* < 0.01) compared to those in the eastern developed zone.

### Utility index

The mean (standard deviation) and median (interquartile range) values of UI scores, stratified by sociodemographic characteristics, are shown in Table 2. The weighted mean UI score was 0.951 (SD = 0.099). Respondents who were male, younger, and those with better behaviours, no chronic conditions, better family conditions, and residence in a relatively economically developed zone had higher UI scores (*p < 0.001*).

The multivariable Tobit regression model indicated that higher UI scores were associated with sleep durations of more than 7 h (β = 0.033, *p* < 0.05), very good sleep quality (β = 0.150, *p* < 0.001), and excellent family functioning (β = 0.056, *p* < 0.001). Conversely, lower UI scores were associated with alcohol consumption (β=-0.051, *p* < 0.001), the presence of chronic conditions (β=-0.146, *p* < 0.001), and having siblings (β=-0.019, *p* < 0.05) (Table 4).

**Table 4:** Factors associated with EQ-5D-Y-3L utility index-results of Tobit regression models (weighted)

	Utility Index (weighted) [Model 1]			Utility Index (weighted) [Model 2]		
Variable	β	SE	95%CI	β	SE	95%CI
Gender						
Ref: Male						
Female	-0.020^*^	0.009	(-0.038, -0.001)	-0.016	0.009	(-0.033, -0.000)
**Age (years)**						
Ref: 8–11						
12–15	-0.043^***^	0.012	(-0.067, -0.019)	-0.017	0.011	(-0.039, 0.006)
16–18	-0.062^***^	0.012	(-0.085, -0.038)	0.001	0.013	(-0.024, 0.026)
**Smoking**						
Ref: No						
Yes				-0.022	0.020	(-0.061, 0.018)
**Drinking**						
Ref: No						
Yes				-0.051^***^	0.009	(-0.070, -0.033)
**Sleep time(hours)**						
Ref: <6						
6–7				0.026	0.016	(-0.004, 0.057)
> 7				0.033^*^	0.016	(0.001, 0.064)
**Sleep quality**						
Ref: Very poor						
poor				0.024	0.029	(-0.033, 0.081)
good				0.095^***^	0.029	(0.038, 0.153)
Very good				0.150^***^	0.030	(0.090, 0.209)
**Chronic conditions**						
Ref: No						
Yes				-0.146^***^	0.017	(-0.180, -0.112)
**BMI**						
Ref: Normal						
Moderate to severe weight loss				-0.021	0.018	(-0.056, 0.014)
Mild weight loss				-0.025	0.020	(-0.065, 0.015)
Overweight				-0.004	0.016	(-0.035, 0.028)
Obesity				-0.015	0.009	(-0.034, 0.003)
**Living arrangement**						
Ref: Living with both parents						
Not living with parents				-0.027	0.015	(-0.056, 0.002)
Living with one parent				-0.017	0.013	(-0.042, 0.009)
**Average family contact per day(hours)**						
Ref: 0–7						
8–15				-0.005	0.012	(-0.028, 0.018)
16–24				-0.008	0.014	(-0.035, 0.019)
**Having siblings**						
Ref: No						
Yes				-0.019^*^	0.009	(-0.037, -0.002)
**Family functioning**						
Ref: Poor						
Moderate				0.009	0.011	(-0.013, 0.030)
Excellent				0.056^***^	0.011	(0.035, 0.078)
**Residence**						
Ref: Urban						
Rural				-0.001	0.009	(-0.019, 0.017)
**Region**						
Ref: Eastern developed						
Central developing				-0.025^**^	0.010	(-0.044, -0.005)
Western underdeveloped				-0.029^**^	0.011	(-0.051, -0.007)
R^2^	0.018			0.251		

*: P-value < 0.05, **: P-value < 0.01, ***: P-value < 0.001,β: standardized beta coefficient in the regression models, SE: standard error, 95% CI: 95% confidence interval

Additionally, respondents residing in the central developing zone (β=-0.025, *p* < 0.01) and the western underdeveloped zone (β=-0.029, *p* < 0.01) had lower UI scores compared to those in the eastern developed region.

There were no significant differences in UI scores based on age, gender, or urban/rural location, after adjustment for variations in other factors.

### Population scores by age, gender and urban - rural distribution

Population norms (weighted) for the EQ-5D-Y-3L are presented for various age groups by urban and rural settings (Appendix Table 2 for urban and Appendix Table 3 for rural), including the percentages of reported problems, as well as the mean (SD, 95% CI) and median (IQR) UI scores. Additionally, population scores by age are reported for male respondents (Appendix Table 4) and female respondents (Appendix Table 5), respectively.

All of the above analyses showed that UI scores decreased with age, and that PD and WSU were the two most frequently reported problem dimensions. In addition, significant urban-rural and gender differences in UI were observed (*p < 0.001*).

### Sensitivity analysis

We conducted sensitivity analyses comparing 5,392 eligible responses (after excluding 103 rejected respondents and 173 individuals under the age of 8 or over 18) with the 5,191 responses presented in the manuscript. The sensitivity analysis demonstrated consistent results (Appendix Table 6).

## Discussion

This study constructed the EQ-5D-Y-3 L norms for Chinese children based on a nationwide survey. It provides a reference for clinicians to evaluate patient-reported health outcomes in children and for public health workers to assess and monitor the health status, treatment outcomes, and healthcare economics of children [41–43].

The weighted average UI score of children in mainland China is 0.951, slightly lower than that reported in the study conducted in Jiangsu province in China [44]. Compared with other countries, China’s UI scores are relatively high: consistent with the 0.90–0.95 reported in Japan [14], slightly lower than 0.99 in Indonesia [17], but higher than 0.88 in Chile [16] and 0.89 in Peru [15].

Overall, the ceiling effect of the EQ-5D-Y-3 L is relatively high in mainland China. About two-thirds (65.38%) of respondents in this study reported no problems in five dimensions (similar to that reported in the Jiangsu study [44]), compared with 56.65% in Indonesia [17], 40% to 60% in Japan [14], 43.98% in Peru [15], and 60% to 95% in Chile [16]. The percentage of respondents in this study reporting problems on the five dimensions of the EQ-5D-Y-3 L is consistently lower compared to other countries. Similar findings were also reported in previous studies of the general Chinese population [13], including in studies using the adult version of EQ-5D [45]. The high ceiling effects observed in China can be attributed to various factors, such as values, culture, and traditions (e.g., tolerance for various health problems) [46, 47], which reflect the influence of traditional Chinese cultural values on health reporting behaviours [48].

Similar to other countries, WSU was the most frequently reported problem. Mental health problems among children appear to be prevalent worldwide. Previous studies have shown that young people have the highest prevalence of mental health disorders compared to individuals at any other stage of the life cycle [49]. This may be due to several reasons, including adolescent vulnerability [50], family life [51], the digital age [52], and the COVID-19 pandemic [53]. However, about 25% of Chinese respondents reported problems with WSU, much less than those from other countries, where the percentage could be as high as 51.7% [16].

The next most frequently reported problems among Chinese children were having pain or discomfort (17.87%) and performing usual activities (7.73%), both of which were also at lower levels than in other countries [14–17]. This might be due to the support of Chinese family structures and the enhancement of a sense of belonging through collectivist activities [54]. In addition, the degree of encouragement for emotional expression varies among different cultures [55]. For example, different cultures have varying understandings and expressions of pain, which may affect the frequency and intensity of children’s pain reports [56]. Furthermore, response styles vary significantly across cultures. For example, in a collectivist culture like China, moderation and harmony are emphasized, leading to a lower usage rate of extreme response options. In contrast, in individualistic cultures, self-expression is encouraged, so extreme responses are more likely to be reported [57]. The support and participation of family members also significantly impact the development of children’s daily activities [58–60]. In Chinese culture, family members typically provide high levels of support to children, reducing problems with daily activities [61].

Gender and age stratifications in EQ-5D-Y-3 L norms are usually required. Overall, male children are less likely to report health problems than female children, while older age is associated with higher odds of reporting health problems according to the findings of this study. These results are consistent with previous studies conducted in China and other countries [62–65]. It is well known that young people face challenges due to major physical, psychological, and behavioral changes [61]. Girls begin to experience hormonal changes of puberty earlier than boys, so girls and boys of the same chronological age may have different experiences [66]. As they age, young people face more stress, such as academic performance, relationship problems, bullying and peer pressure, financial issues, emerging adult responsibilities, and worries about the future [67, 68].

There are no significant differences in the EQ-5D-Y-3 L UI scores across age, gender, and urban-rural locations, according to the multivariable regression analyses in this study. The high ceiling effects may have limited our ability to distinguish subtle differences. However, regional disparities in HRQoL among Chinese children are evident according to the findings of this study and others [45]. Those residing in the eastern, more developed zones have higher HRQoL than their counterparts in the central developing and western underdeveloped zones. Rural respondents in this study were also more likely to report health problems, although no significant urban-rural differences were found in UI scores. There is ample evidence that urban-rural health disparities in China are mainly due to socioeconomic and medical resource inequalities [69–72].

Family is an important source of social support for children. We found that family functioning is positively associated with UI scores, although living with parents is not. This may reflect the partial compensatory role of extended families (such as grandparents) in China, but we should not ignore the long-term risks to mental health posed by the absence of parental emotional support [73]. Parents in China often impose stricter requirements on the academic performance of their children than other family members, which may weaken the health-protective effect of parental companionship [74]. We also found that families with multiple children saw fewer problems in the self-care abilities of children, but more problems in mental health. This requires further investigation as China has moved away from its one-child family planning policy [75].

This study also offers some insights into other predictors of HRQoL in children. Alcohol consumption, insufficient sleep, poor sleep quality, and chronic conditions are all associated with lower HRQoL as measured by the EQ-5D-Y-3 L. These results are consistent with the findings of previous studies [76–80], highlighting potential measures for improving HRQoL.

This study has several limitations worth noting. Firstly, significant regional disparities exist in China. Populations in different regions may have different preferences and tolerance for various health conditions, but the limited sample size and data availability in this study prevent us from providing province-specific scores. The value set for calculating UI scores also derived from a selected sample of only eight regions [19]. Secondly, study participants were recruited using multiple strategies across various sites, which may help ensure diversity among participants but does not necessarily guarantee representativeness. The literacy level of child participants and the use of an online survey platform may influence the accuracy of the data entered. Although assistance from guardians and investigators may help child participants, their involvement could affect the reliability of the children’s responses. Moreover, MLICC recruited participants from schools, residential communities, and hospitals. Unfortunately, we were unable to perform subgroup analyses by setting due to the unavailability of data. The data did not capture information about which participants were unable to use the online platform or the proportion of this group. However, digital tools are widely used in China, especially among younger generations.

## Conclusion

This study provides national EQ-5D-Y-3L population norms for children in mainland China, including overall and stratified UI scores by age, gender, and urban-rural location. The study found that the overall HRQoL of Chinese children is at a relatively high level, with scores comparable to those of Japan. Although slightly lower than that of Indonesia, it is significantly higher than that of Chile and Peru. Anxiety/depression is the most commonly reported health problem in children in mainland China, followed by pain/discomfort. A wide range of socioeconomic and behavioral factors are associated with EQ-5D-Y-3L scores.

## Supplementary Information

Below is the link to the electronic supplementary material.

Supplementary Material 1

Supplementary Material 2

Supplementary Material 3

Supplementary Material 4

Supplementary Material 5

Supplementary Material 6

## Data Availability

Data are available on reasonable request. Data are not publicly available but may be requested from the authors on reasonable request. Data used in this study were extracted from the Medication Literacy Investigation of Chinese Children (MLICC) conducted by the author Yibo Wu.
