# Supplementary material for: EQ-5D-Y-3L population norms for children in Mainland China derived from a national survey 2023–2024
Source: Health Qual Life Outcomes. 2025 Dec 29;24:15. doi: 10.1186/s12955-025-02470-z (PMC12860117; doi:10.1186/s12955-025-02470-z)
Supplement: Supplementary file 1 — Supplementary Material 1 [file 12955_2025_2470_MOESM1_ESM.docx]

| **S1. Composition of the sample population compared to the composition of the 2020 China Population Census (%)** | | | | | | | |
| --- | --- | --- | --- | --- | --- | --- | --- |
| **Variables** | | | **MLICC** |  | **The 2020 China Population Census** | | **Weight** |
| **Residence** | Gender | Age | **N** | **%** | **N** | **%** |  |
| **Urban** | Male | 8-11 | 485 | 9.34% | 12900922 | 11.24% | 1.203 |
|  |  | 12-15 | 696 | 13.41% | 11621310 | 10.12% | 0.755 |
|  |  | 16-18 | 454 | 8.75% | 10275397 | 8.95% | 1.024 |
|  |  |  | 1635 | 31.50% | 34797629 | 30.31% | 0.962 |
|  | Female | 8-11 | 423 | 8.15% | 11218212 | 9.77% | 1.199 |
|  |  | 12-15 | 698 | 13.45% | 10174410 | 8.86% | 0.659 |
|  |  | 16-18 | 734 | 14.14% | 9054198 | 7.89% | 0.558 |
|  |  |  | 1855 | 35.73% | 30446820 | 26.52% | 0.742 |
| **Rural** | Male | 8-11 | 116 | 2.23% | 10140085 | 8.83% | 3.953 |
|  |  | 12-15 | 340 | 6.55% | 9476524 | 8.26% | 1.260 |
|  |  | 16-18 | 273 | 5.26% | 6845495 | 5.96% | 1.134 |
|  |  |  | 729 | 14.04% | 26462104 | 23.05% | 1.642 |
|  | Female | 8-11 | 80 | 1.54% | 8754909 | 7.63% | 4.949 |
|  |  | 12-15 | 343 | 6.61% | 8276850 | 7.21% | 1.091 |
|  |  | 16-18 | 549 | 10.58% | 6050624 | 5.27% | 0.498 |
|  |  |  | 972 | 18.72% | 23082383 | 20.11% | 1.074 |
|  |  | Total | 5191 | 1 | 114788936 | 1 |  |
