# Supplementary material for: EQ-5D-Y-3L population norms for children in Mainland China derived from a national survey 2023–2024
Source: Health Qual Life Outcomes. 2025 Dec 29;24:15. doi: 10.1186/s12955-025-02470-z (PMC12860117; doi:10.1186/s12955-025-02470-z)
Supplement: Supplementary file 2 — Supplementary Material 2 [file 12955_2025_2470_MOESM2_ESM.docx]

| **S2. Percentage of study participants reporting problems and EQ-5D-Y-3L utility index scores by age group for urban (weighted)** | | | | | |
| --- | --- | --- | --- | --- | --- |
| **EQ-5D-Y-3L dimension** |  | **8-11** | **12-15** | **16-18** | **Total** |
|  |  | N=1061 | N=1018 | N=871 | N=2951 |
| **Mobility** | No problems | 97.25% | 96.53% | 96.82% | 96.87% |
|  | Some problems | 1.65% | 2.82% | 2.82% | 2.40% |
|  | Extreme problems | 1.10% | 0.65% | 0.36% | 0.73% |
|  | *P value* | ***p<0.001*** | | |  |
| **Looking after myself** | No problems | 94.27% | 97.37% | 98.48% | 96.58% |
|  | Some problems | 4.63% | 2.05% | 1.05% | 2.68% |
|  | Extreme problems | 1.10% | 0.58% | 0.47% | 0.74% |
|  | *P value* | ***p<0.001*** | | |  |
| **Doing usual activities** | No problems | 93.94% | 93.51% | 92.78% | 93.45% |
|  | Some problems | 5.40% | 5.98% | 6.62% | 5.96% |
|  | Extreme problems | 0.66% | 0.51% | 0.60% | 0.59% |
|  | *P value* | ***p<0.001*** | | |  |
| **Having pain or discomfort** | No problems | 89.10% | 80.41% | 78.89% | 83.09% |
|  | Some problems | 10.24% | 18.37% | 20.46% | 16.06% |
|  | Extreme problems | 0.66% | 1.22% | 0.65% | 0.85% |
|  | *P value* | ***p<0.001*** | | |  |
| **Feeling worried, sad or unhappy** | No problems | 84.69% | 73.43% | 68.92% | 76.15% |
|  | Some problems | 14.76% | 22.87% | 27.84% | 21.42% |
|  | Extreme problems | 0.55% | 3.70% | 3.24% | 2.43% |
|  | *P value* | ***p<0.001*** | | |  |
| **Utility index** | Mean | 0.969 | 0.950 | 0.948 | 0.956 |
|  | SD | 0.077 | 0.096 | 0.090 | 0.088 |
|  | 95%CI | (0.967,0.970) | (0.949,0.952) | (0.946,0.950) | (0.955,0.957) |
|  | Median | 1.000 | 1.000 | 1.000 | 1.000 |
|  | IQR | 0.023 | 0.073 | 0.073 | 0.073 |
|  | 25th percentile | 0.977 | 0.927 | 0.927 | 0.927 |
|  | 75th percentile | 1.000 | 1.000 | 1.000 | 1.000 |
|  | *P value* | ***p<0.001*** | | |  |
