# Supplementary material for: EQ-5D-Y-3L population norms for children in Mainland China derived from a national survey 2023–2024
Source: Health Qual Life Outcomes. 2025 Dec 29;24:15. doi: 10.1186/s12955-025-02470-z (PMC12860117; doi:10.1186/s12955-025-02470-z)
Supplement: Supplementary file 3 — Supplementary Material 3 [file 12955_2025_2470_MOESM3_ESM.docx]

| **S3. Percentage of study participants reporting problems and EQ-5D-Y-3L utility index scores by age group for rural (weighted)** | | | | | |
| --- | --- | --- | --- | --- | --- |
| **EQ-5D-Y-3L dimension** |  | **8-11** | **12-15** | **16-18** | **Total** |
|  |  | N=852 | N=816 | N=572 | N=2240 |
| **Mobility** | No problems | 92.31% | 94.35% | 92.43% | 93.08% |
|  | Some problems | 5.25% | 4.37% | 5.86% | 5.08% |
|  | Extreme problems | 2.44% | 1.28% | 1.70% | 1.83% |
|  | *P value* | ***p<0.001*** | | |  |
| **Looking after myself** | No problems | 92.68% | 96.06% | 95.89% | 94.73% |
|  | Some problems | 6.86% | 2.77% | 3.64% | 4.55% |
|  | Extreme problems | 0.47% | 1.17% | 0.47% | 0.72% |
|  | *P value* | ***p<0.001*** | | |  |
| **Doing usual activities** | No problems | 91.64% | 90.32% | 89.84% | 90.70% |
|  | Some problems | 6.86% | 7.76% | 9.22% | 7.79% |
|  | Extreme problems | 1.51% | 1.92% | 0.94% | 1.51% |
|  | *P value* | ***p<0.001*** | | |  |
| **Having pain or discomfort** | No problems | 83.89% | 94.35% | 75.99% | 80.66% |
|  | Some problems | 13.56% | 17.94% | 22.25% | 17.38% |
|  | Extreme problems | 2.55% | 1.49% | 1.76% | 1.96% |
|  | *P value* | ***p<0.001*** | | |  |
| **Feeling worried, sad or unhappy** | No problems | 80.09% | 73.67% | 66.18% | 74.20% |
|  | Some problems | 15.18% | 23.34% | 30.55% | 22.07% |
|  | Extreme problems | 4.73% | 2.99% | 3.27% | 3.72% |
|  | *P value* | ***p<0.001*** | | |  |
| **Utility index** | Mean | 0.947 | 0.945 | 0.936 | 0.943 |
|  | SD | 0.120 | 0.108 | 0.103 | 0.111 |
|  | 95%CI | (0.945,0.950) | (0.942,0.947) | (0.933,0.938) | (0.942,0.945) |
|  | Median | 1.000 | 1.000 | 1.000 | 1.000 |
|  | IQR | 0.073 | 0.073 | 0.090 | 0.073 |
|  | 25th percentile | 0.927 | 0.927 | 0.910 | 0.927 |
|  | 75th percentile | 1.000 | 1.000 | 1.000 | 1.000 |
|  | *P value* | ***p<0.001*** | | |  |
