# Supplementary material for: EQ-5D-Y-3L population norms for children in Mainland China derived from a national survey 2023–2024
Source: Health Qual Life Outcomes. 2025 Dec 29;24:15. doi: 10.1186/s12955-025-02470-z (PMC12860117; doi:10.1186/s12955-025-02470-z)
Supplement: Supplementary file 4 — Supplementary Material 4 [file 12955_2025_2470_MOESM4_ESM.docx]

| **S4. Percentage of study participants reporting problems and EQ-5D-Y-3L utility index scores by age group for males (weighted)** | | | | | |
| --- | --- | --- | --- | --- | --- |
| **EQ-5D-Y-3L dimension** |  | **8-11** | **12-15** | **16-18** | **Total** |
|  |  | N=1035 | N=988 | N=746 | N=2770 |
| **Mobility** | No problems | 92.58% | 95.62% | 93.77% | 93.99% |
|  | Some problems | 5.09% | 3.58% | 4.80% | 4.47% |
|  | Extreme problems | 2.33% | 0.79% | 1.43% | 1.54% |
|  | *P value* | ***p<0.001*** | | |  |
| **Looking after myself** | No problems | 92.54% | 96.47% | 96.74% | 95.07% |
|  | Some problems | 6.39% | 2.61% | 2.57% | 4.01% |
|  | Extreme problems | 1.07% | 0.92% | 0.69% | 0.91% |
|  | *P value* | ***p<0.001*** | | |  |
| **Doing usual activities** | No problems | 91.59% | 93.07% | 90.93% | 91.94% |
|  | Some problems | 7.07% | 5.70% | 8.09% | 6.86% |
|  | Extreme problems | 1.34% | 1.23% | 0.98% | 1.20% |
|  | *P value* | ***p<0.001*** | | |  |
| **Having pain or discomfort** | No problems | 85.77% | 83.03% | 81.24% | 83.57% |
|  | Some problems | 12.62% | 15.47% | 17.75% | 15.02% |
|  | Extreme problems | 1.61% | 1.50% | 1.01% | 1.41% |
|  | *P value* | ***p<0.001*** | | |  |
| **Feeling worried, sad or unhappy** | No problems | 82.83% | 77.81% | 73.56% | 78.54% |
|  | Some problems | 15.30% | 19.43% | 23.08% | 18.87% |
|  | Extreme problems | 1.87% | 2.75% | 3.35% | 2.59% |
|  | *P value* | ***p<0.001*** | | |  |
| **Utility index** | Mean | 0.955 | 0.954 | 0.948 | 0.953 |
|  | SD | 0.103 | 0.100 | 0.102 | 0.102 |
|  | 95%CI | (0.953,0.957) | (0.952,0.956) | （0.945,0.950） | (0.951,0.954) |
|  | Median | 1.000 | 1.000 | 1.000 | 1.000 |
|  | IQR | 0.069 | 0.073 | 0.073 | 0.073 |
|  | 25th percentile | 0.931 | 0.927 | 0.927 | 0.927 |
|  | 75th percentile | 1.000 | 1.000 | 1.000 | 1.000 |
|  | *P value* | ***p<0.001*** | | |  |
