# Supplementary material for: EQ-5D-Y-3L population norms for children in Mainland China derived from a national survey 2023–2024
Source: Health Qual Life Outcomes. 2025 Dec 29;24:15. doi: 10.1186/s12955-025-02470-z (PMC12860117; doi:10.1186/s12955-025-02470-z)
Supplement: Supplementary file 5 — Supplementary Material 5 [file 12955_2025_2470_MOESM5_ESM.docx]

| **S5. Percentage of study participants reporting problems and EQ-5D-Y-3L utility index scores by age group for females (weighted)** | | | | | |
| --- | --- | --- | --- | --- | --- |
| **EQ-5D-Y-3L dimension** |  | **8-11** | **12-15** | **16-18** | **Total** |
|  |  | N=878 | N=845 | N=698 | N=2421 |
| **Mobility** | No problems | 97.98% | 95.51% | 96.52% | 96.69% |
|  | Some problems | 1.08% | 3.41% | 3.18% | 2.50% |
|  | Extreme problems | 0.94% | 1.09% | 0.31% | 0.81% |
|  | *P value* | ***p<0.001*** | | |  |
| **Looking after myself** | No problems | 94.76% | 97.17% | 98.24% | 96.61% |
|  | Some problems | 4.70% | 2.08% | 1.52% | 2.87% |
|  | Extreme problems | 0.53% | 0.75% | 0.24% | 0.52% |
|  | *P value* | ***p<0.001*** | | |  |
| **Doing usual activities** | No problems | 94.49% | 91.00% | 92.36% | 92.66% |
|  | Some problems | 4.84% | 7.99% | 7.16% | 6.61% |
|  | Extreme problems | 0.68% | 1.00% | 0.48% | 0.73% |
|  | *P value* | ***p<0.001*** | | |  |
| **Having pain or discomfort** | No problems | 88.01% | 77.54% | 74.08% | 80.34% |
|  | Some problems | 10.64% | 21.31% | 24.76% | 18.43% |
|  | Extreme problems | 1.36% | 1.15% | 1.16% | 1.23% |
|  | *P value* | ***p<0.001*** | | |  |
| **Feeling worried, sad or unhappy** | No problems | 82.48% | 68.60% | 61.81% | 71.68% |
|  | Some problems | 14.53% | 27.28% | 35.04% | 24.89% |
|  | Extreme problems | 2.99% | 4.12% | 3.15% | 3.43% |
|  | *P value* | ***p<0.001*** | | |  |
| **Utility index** | Mean | 0.964 | 0.941 | 0.938 | 0.949 |
|  | SD | 0.093 | 0.102 | 0.088 | 0.096 |
|  | 95%CI | (0.962,0.966) | (0.939,0.943) | (0.936,0.940) | (0.947,0.950) |
|  | Median | 1.000 | 1.000 | 1.000 | 1.000 |
|  | IQR | 0.023 | 0.084 | 0.090 | 0.073 |
|  | 25th percentile | 0.977 | 0.916 | 0.910 | 0.927 |
|  | 75th percentile | 1.000 | 1.000 | 1.000 | 1.000 |
|  | *P value* | ***p<0.001*** | | |  |
