# Supplementary material for: EQ-5D-Y-3L population norms for children in Mainland China derived from a national survey 2023–2024
Source: Health Qual Life Outcomes. 2025 Dec 29;24:15. doi: 10.1186/s12955-025-02470-z (PMC12860117; doi:10.1186/s12955-025-02470-z)
Supplement: Supplementary file 6 — Supplementary Material 6 [file 12955_2025_2470_MOESM6_ESM.docx]

| **S6. Sensitivity analysis - Factors associated with EQ-5D-Y-3L utility index-results of Tobit regression models (weighted) using the sample containing 5,392 eligible responses** | | | | | | |
| --- | --- | --- | --- | --- | --- | --- |
|  | **Utility Index (weighted) [Model 1]** | | | **Utility Index (weighted) [Model 2]** | | |
| **Variable** | **β** | **SE** | **95%CI** | **β** | **SE** | **95%CI** |
| **Gender** |  |  |  |  |  |  |
| Ref: Male |  |  |  |  |  |  |
| Female | -0.021* | 0.010 | (-0.040，-0.002) | -0.016 | 0.008 | (-0.032, 0.001) |
| **Age (years)** |  |  |  |  |  |  |
| Ref: 8-11 |  |  |  |  |  |  |
| 12-15 | -0.038** | 0.012 | (-0.062，-0.014) | -0.013 | 0.012 | (-0.037, 0.010) |
| 16-18 | -0.054*** | 0.013 | (-0.078，-0.030) | 0.010 | 0.014 | (-0.018, 0.038) |
| **Smoking** |  |  |  |  |  |  |
| Ref: No |  |  |  |  |  |  |
| Yes |  |  |  | -0.044 | 0.025 | (-0.094, 0.005) |
| **Drinking** |  |  |  |  |  |  |
| Ref: No |  |  |  |  |  |  |
| Yes |  |  |  | -0.047*** | 0.010 | (-0.067, -0.027) |
| **Sleep time(hours)** |  |  |  |  |  |  |
| Ref: <6 |  |  |  |  |  |  |
| 6-7 |  |  |  | 0.016 | 0.015 | (-0.014, 0.046) |
| >7 |  |  |  | 0.027 | 0.016 | (-0.003, 0.058) |
| **Sleep quality** |  |  |  |  |  |  |
| Ref: Very poor |  |  |  |  |  |  |
| poor |  |  |  | 0.020 | 0.027 | (-0.034, 0.073) |
| good |  |  |  | 0.087*** | 0.027 | (0.034, 0.141) |
| Very good |  |  |  | 0.149*** | 0.029 | (0.093, 0.205) |
| **Chronic conditions** |  |  |  |  |  |  |
| Ref: No |  |  |  |  |  |  |
| Yes |  |  |  | -0.150*** | 0.017 | (-0.184, -0.117) |
| **BMI** |  |  |  |  |  |  |
| Ref: Normal |  |  |  |  |  |  |
| Moderate to severe weight loss |  |  |  | -0.002 | 0.017 | (-0.035, 0.030) |
| Mild weight loss |  |  |  | 0.000 | 0.019 | (-0.038, 0.037) |
| Overweight |  |  |  | -0.011 | 0.016 | (-0.043, 0.021) |
| Obesity |  |  |  | -0.004 | 0.014 | (-0.032, 0.023) |
| **Living arrangement** |  |  |  |  |  |  |
| Ref: Living with both parents |  |  |  |  |  |  |
| Not living with parents |  |  |  | -0.027* | 0.014 | (-0.055, 0.001) |
| Living with one parent |  |  |  | -0.019 | 0.013 | (-0.043, 0.006) |
| **Average family contact per day(hours)** |  |  |  |  |  |  |
| Ref: 0-7 |  |  |  |  |  |  |
| 8-15 |  |  |  | -0.011 | 0.012 | (-0.034,0.012) |
| 16-24 |  |  |  | -0.008 | 0.013 | (-0.033,0.018) |
| **Having siblings** |  |  |  |  |  |  |
| Ref: No |  |  |  |  |  |  |
| Yes |  |  |  | -0.023* | 0.010 | (-0.041, -0.004) |
| **Family health** |  |  |  |  |  |  |
| Ref: Poor |  |  |  |  |  |  |
| Moderate |  |  |  | 0.015* | 0.011 | (-0.006, 0.036) |
| Excellent |  |  |  | 0.056* | 0.012 | (0.033, 0.080) |
| **Residence** |  |  |  |  |  |  |
| Ref: Urban |  |  |  |  |  |  |
| Rural |  |  |  | 0.006 | 0.010 | (-0.013, 0.025) |
| **Region** |  |  |  |  |  |  |
| Ref: Eastern developed |  |  |  |  |  |  |
| Central developing |  |  |  | -0.012 | 0.011 | (-0.034, 0.010) |
| Western underdeveloped |  |  |  | -0.025* | 0.012 | (-0.048, -0.001) |
| R^2^ | 0.013 | | | 0.242 | | |
| *: P-value< 0.05, **: P-value< 0.01, ***: P-value < 0.001,β: standardized beta coefficient in the regression models, SE : standard error, 95% CI : 95% confidence interval. | | | | | | |
